# Perforator preservation technologies (PPT) based on a new neuro-interventional classification in endovascular treatment of perforator involving aneurysms (piANs)

**DOI:** 10.1186/s41016-021-00243-3

**Published:** 2021-05-02

**Authors:** Chen Li, Ao-Fei Liu, Han-Cheng Qiu, Xianli Lv, Ji Zhou, Yi-Qun Zhang, Jin Lv, Ying-Ying Zhang, Sushan Hu, Fang Liu, Yun-e Liu, Min Jin, Wei-Jian Jiang

**Affiliations:** 1 New Era Stroke Care and Research Institute, PLA Rocket Force Characteristic Medical Center, 18 Xinjiekouwai Street, Beijing, 100088 China; 2 Neurosurgery Department, Beijing Tsinghua Changgung Hospital, School of Clinical Medicine, Tsinghua University, Beijing, China

**Keywords:** Intracranial aneurysm, Classification, Perforator, Endovascular treatment, Stroke, Subarachnoid hemorrhage

## Abstract

**Background:**

Treatment of perforator involving aneurysm (piAN) remains a challenge to open and endovascular neurosurgeons. Our aim is to demonstrate a primary outcome of endovascular therapy for piANs with the use of perforator preservation technologies (PPT) based on a new neuro-interventional classification.

**Methods:**

The piANs were classified into type I: aneurysm really arises from perforating artery, type II: saccular aneurysm involves perforating arteries arising from its neck (IIa) or dome (IIb), and type III: fusiform aneurysm involves perforating artery. Stent protection technology of PPT was applied in type I and III aneurysms, and coil-basket protection technology in type II aneurysms. An immediate outcome of aneurysmal obliteration after treatment was evaluated (satisfactory obliteration: the saccular aneurysm body is densely embolized (I), leaving a gap in the neck (IIa) or dome (IIb) where the perforating artery arising; fusiform aneurysm is repaired and has a smooth inner wall), and successful perforating artery preservation was defined as keeping the good antegrade flow of those perforators on postoperative angiography. The periprocedural complication was closely monitored, and clinical and angiographic follow-ups were performed.

**Results:**

Six consecutive piANs (2 ruptured and 4 unruptured; 1 type I, 2 type IIa, 2 type IIb, and 1 type III) in 6 patients (aged from 43 to 66 years; 3 males) underwent endovascular therapy between November 2017 and July 2019. The immediate angiography after treatment showed 6 aneurysms obtained satisfactory obliteration, and all of their perforating arteries were successfully preserved. During clinical follow-up of 13–50 months, no ischemic or hemorrhagic event of the brain occurred in the 6 patients, but has one who developed ischemic event in the territory of involving perforators 4 h after operation and completely resolved within 24 h. Follow-up angiography at 3 to 10M showed patency of the parent artery and perforating arteries of treated aneurysms, with no aneurysmal recurrence.

**Conclusions:**

Our perforator preservation technologies on the basis of the new neuro-interventional classification seem feasible, safe, and effective in protecting involved perforators while occluding aneurysm.

## Background

The standard methods of treatment for intracranial aneurysms are surgical clipping and endovascular treatment, both of which are very mature [1, 2]. However, treatment of perforator involving aneurysm (piAN) remains a challenge to open and endovascular neurosurgeons [3, 4], because the success of aneurysm surgeries lies in the complete clipping of the aneurysm neck and in the preservation of branching and perforating arteries [3]. The same is true for endovascular treatment of intracranial aneurysms. Injury to perforating arteries has always been one of the major causes of postoperative morbidity in aneurysm treatment [4]. Under the premise of ensuring complete treatment of aneurysm neck, better techniques are needed to protect the perforators. Researchers try to classify piANs and formulate corresponding treatments to improve the success rate of surgery. Satti et al. proposed a three-point classification based on the exact anatomical origin of basilar artery perforator aneurysms (BAPAs) and present this unique classification system to enable future papers to standardize descriptions: type I—the aneurysm arises from the basilar trunk adjacent to the perforating arterial branch but not involving a perforating artery; type IIa—aneurysms incorporating the origin of the perforating arteries; type IIb—aneurysms having the perforating artery arising from the dome of the aneurysm; and type III—fusiform aneurysms arising beyond the parent vessel (basilar artery) [3, 4]. However, this classification method is only applicable to BAPAs, not completely applicable to all intracranial piANs. We improved the classification method based on the characteristics and feasibility of interventional therapy and also proposed some perforator preservation technologies (PPT) on basis of this classification, which can protect the blood supply of the perforator artery on the premise of ensuring the satisfactory aneurysm packing. Here, we report the preliminary treatment results.

## Methods

### Patient population

From November 2017 to July 2019, 6 consecutive patients with 6 piANs received endovascular treatment in our center. All of the demographic data, clinical presentation, aneurysm size and location, therapeutic intervention, immediate angiographic, and clinical result, as well as clinical and radiological follow-up information were recorded and analyzed.

**Fig. 1:**
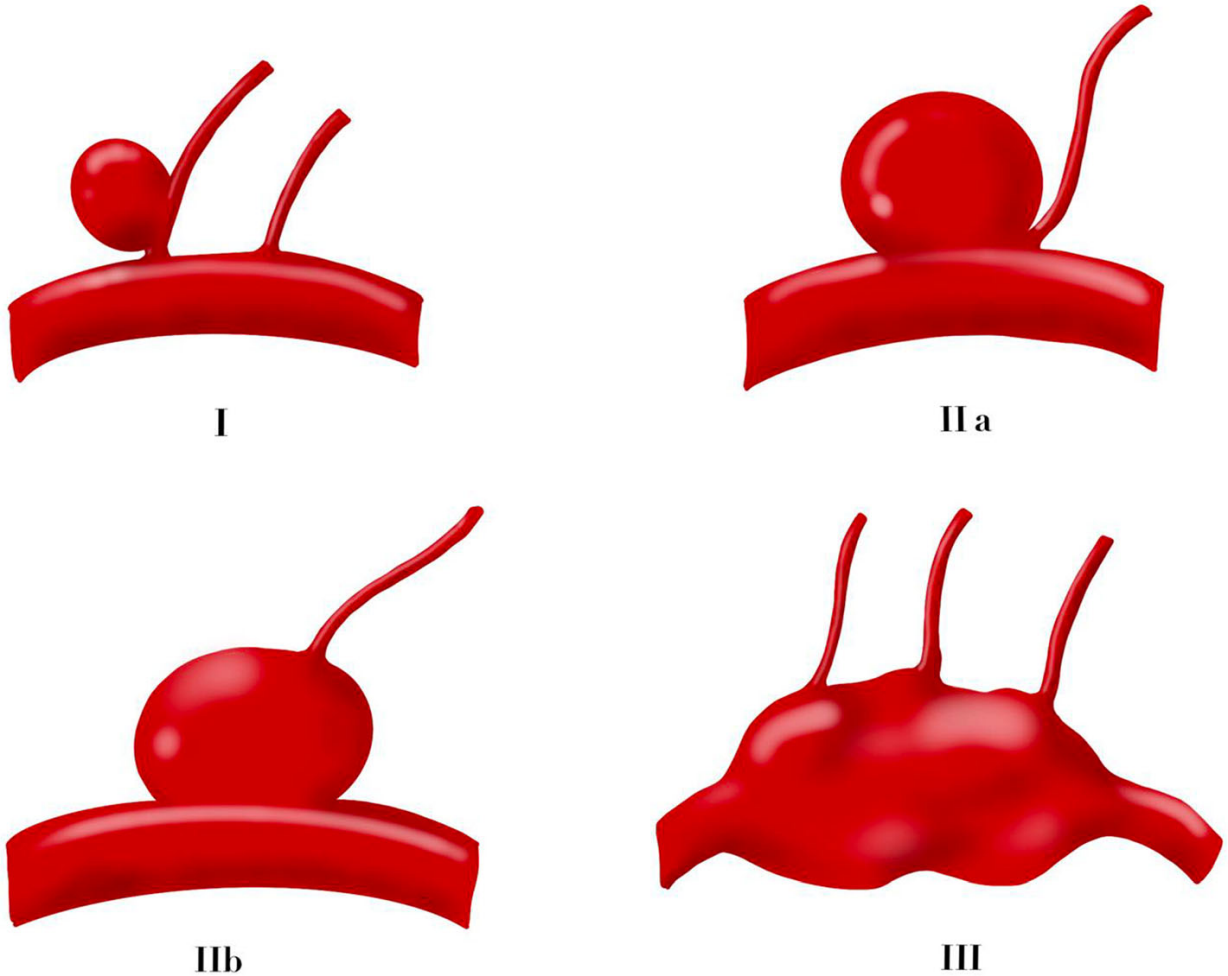
Our unique three-point classification based on the anatomy relationship between aneurysms and perforating arteries: type I—aneurysm arises from the perforating artery; type IIa—aneurysms having the perforating artery arising from the neck of the aneurysm; type IIb—aneurysms having the perforating artery arising from the dome of the aneurysm; and type III—fusiform aneurysms involve all trunk and perforating branches of the parent artery

We proposed a three-point classification based on the anatomy relationship between aneurysms and perforating arteries after summarizing the characteristics of these piANs (Fig. 1):

- Type I—aneurysm arises from the perforating artery
- Type IIa—aneurysms having the perforating artery arising from the neck of the aneurysm
- Type IIb—aneurysms having the perforating artery arising from the dome of the aneurysm
- Type III—fusiform aneurysms involve all trunk and perforating branches of the parent artery

### Endovascular treatment

For patients with unruptured intracranial aneurysms, their pre-medication was given oral doses of aspirin (100 mg) and Clopidogrel (75 mg) every morning for at least 4 days prior to the operation. For patients with SAH, we do not give antiplatelet drugs before surgery. If a stent needs to be implanted during the operation, aspirin 300mg + Clopidogrel 300mg is given through a nasogastric tube at one time. The post-procedural antiplatelet regimen is unified, consisted of aspirin (100 mg once daily) and Clopidogrel (75 mg once daily) continued for 3 months following treatment and aspirin (100 mg once daily) continued for life.

All 6 patients received intravascular treatment and all the treatments were performed under general anesthesia. All procedures were performed via the right common femoral route using a 6Fr access system as standard. All procedures were performed under heparin anticoagulation with a 3000 IU bolus dose at the start of the procedure and subsequent 1000 IU bolus doses every hour to maintain the activated clotting time between 2 and 2.5 times the baseline. The selection of stents and coils was based on operator preference as well as the size of the aneurysms and parent vessels.

Based on the abovementioned aneurysm classification, we summarized two major types of perforator preservation technologies (PPT). It is mainly a coil-basket protection technology, and the other is a stent protection technology. Different protection techniques were used to develop a personalized surgical plan.

Type I aneurysms arise from the perforating artery. However, the diameter of the perforator is usually very small, and it is not easy to release the stent in the perforator. We can place a braided stent on the main parent vessel and push the stent when it is adjacent to the perforating artery, which is commonly called the lantern technique to protect the starting lumen of the perforating artery.

Type IIa aneurysms having the perforating artery arising from the neck. If the neck of the aneurysm is densely packed conventionally, the perforating artery will definitely be affected. We choose different diameters coils to densely embolize the aneurysm cavity, and the aneurysm neck near the perforator originating is loosely filled, leaving a little space to ensure blood supply to the perforating artery.

Type IIb aneurysms have the perforating artery arising from the dome of the aneurysm. If all the aneurysm body is packed densely, it will lead to occlusion of the perforating artery. We still use different diameters coils to embolize the main part of the aneurysm body, leaving a certain space at the dome of the aneurysm.

Type III fusiform aneurysms involve all trunk and perforating branches of the parent artery. We implant multiple stents in dissection to block blood flow as much as possible while protecting the perforating artery.

### Procedural assessment and follow-up

Usually, the Raymond–Roy Occlusion Classification (RROC) qualitatively assesses intracranial aneurysm occlusion following endovascular coil embolization. The Modified Raymond–Roy Classification (MRRC) was developed as a refinement of this classification scheme (Class I: complete obliteration; Class II: residual neck; Class IIIa: residual aneurysm has contrast within the coil interstices; Class IIIb: contrast along the aneurysm wall) [5, 6]. However, MRRC does not seem to be suitable for the assessment of satisfactory embolization of perforator involving aneurysms. Here, we propose a new definition of satisfactory obliteration based on our new classification and perforator preservation technologies (PPT) for perforator involving aneurysms:

- Type I—the whole saccular aneurysm body is densely embolized which arises from the perforating artery;
- Type IIa—the saccular aneurysm body is densely embolized leaving a gap in the neck where the perforating artery arising from;
- Type IIb—the saccular aneurysm body is densely embolized leaving a gap in the dome where the perforating artery arising from;
- Type III—fusiform aneurysm is repaired and has a smooth inner wall.

Immediate outcome of aneurysmal obliteration after treatment was evaluated. Patency and flow characteristics within the aneurysm and parent artery were also assessed immediately after treatment of the aneurysms and during follow-up. Successful perforating artery preservation was defined as keeping good antegrade flow of those perforators on postoperative angiography. All of the complications during the perioperative period, clinical data during follow-up, and imaging results were recorded and analyzed. Procedural follow-up was performed initially at 1–3 months, again at 6–12 months, and then once per year. Standard angiographic projections were used to assess the patency of the vessels and the aneurysms.

## Results

**Fig. 2:**
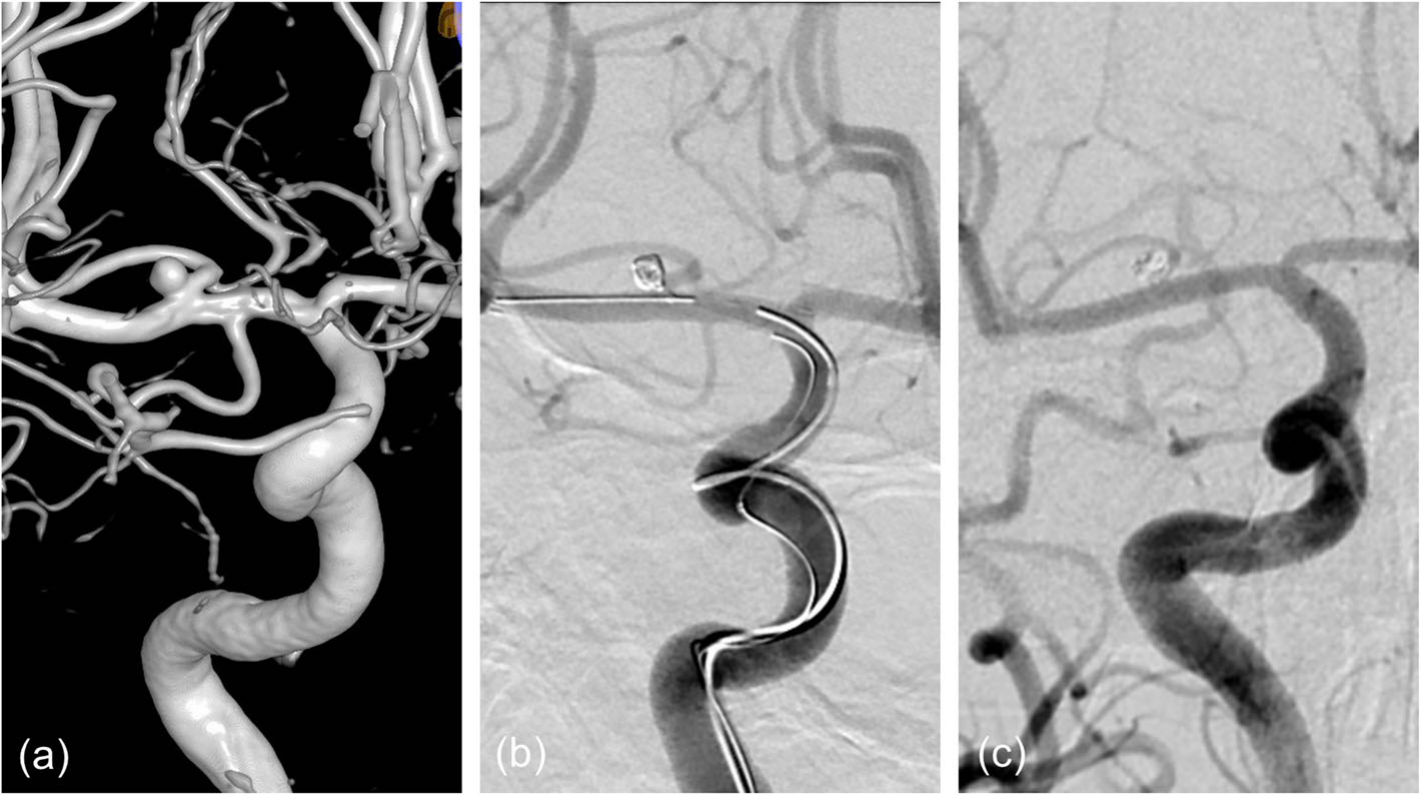
**a** The angiography of patient 4 showed a small aneurysm located at one of the lenticulostriate arteries. **b** We used small coils to embolize the aneurysm and planted a stent on the RM1. When the stent is adjacent to the aneurysm, we pushed it gently ensure the blood supply of the lenticulostriate artery. Postoperative angiography showed satisfactory embolization of the aneurysm and perfect preservation of the lenticulostriate artery (yellow arrow). **c** Follow-up angiography after 9 months showed no aneurysmal recurrence and perfect preservation of the lenticulostriate artery (yellow arrow)

**Fig. 3:**
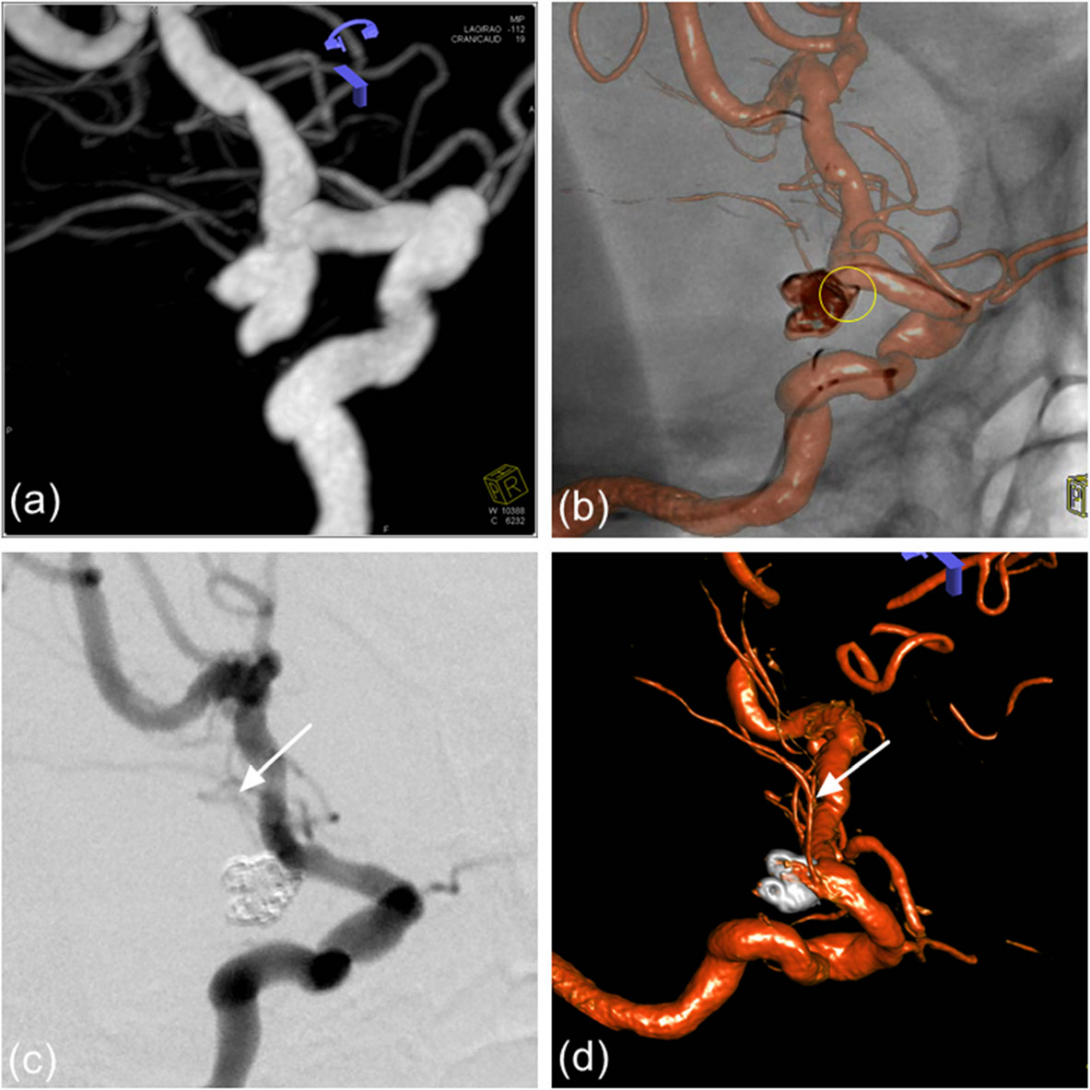
**a** The angiography of patient 1 showed a lobulated LC7 aneurysm with the anterior choroidal artery origin from its neck. **b** According to the measured diameter, different coils are used to densely embolize the two parts of the aneurysm respectively. Leave a little space (yellow circle) at the neck to ensure blood supply to the anterior choroidal artery. **c** Postoperative angiography showed satisfactory embolization of the aneurysm and perfect preservation of anterior choroidal artery (white arrow). **d** Follow-up angiography after 3 months showed no aneurysmal recurrence and perfect preservation of the lenticulostriate artery (white arrow)

**Fig. 4:**
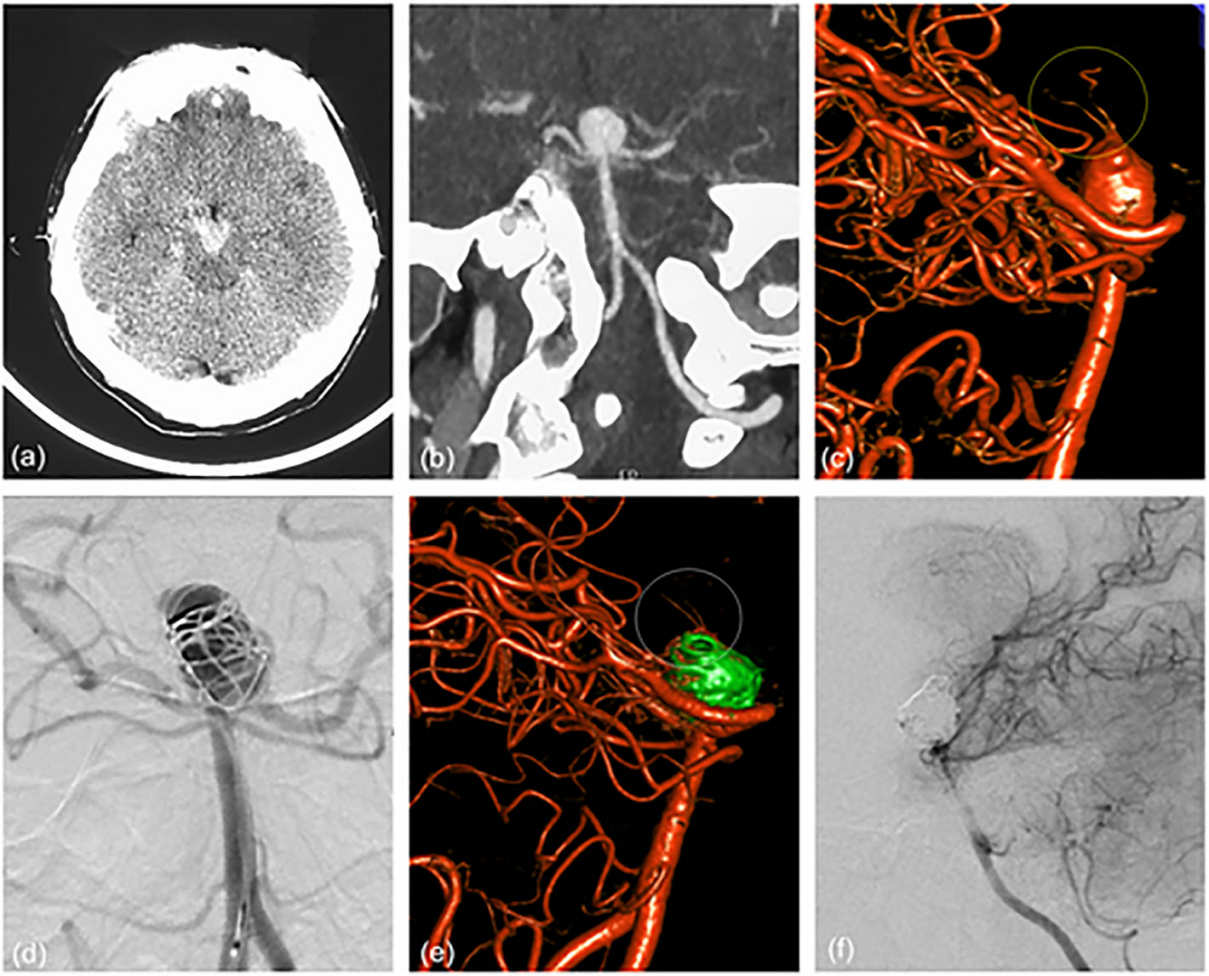
**a**, **b** Patient 2 presented with acute SAH and the initial CTA showed a huge aneurysm at the tip of the basilar artery. **c** The 3D rotational angiogram showed many perforating arteries on the dome of the aneurysm (yellow circle). **d** We used different diameters coils to embolize the main part of the aneurysm body, leaving a certain space at the dome of the aneurysm. **e** Postoperative angiography showed satisfactory embolization of the aneurysm and perfect preservation of perforating arteries on the dome of the aneurysm (white circle). **f** Follow-up angiography after 4 months showed no aneurysmal recurrence and perfect preservation of the lenticulostriate artery

**Fig. 5:**
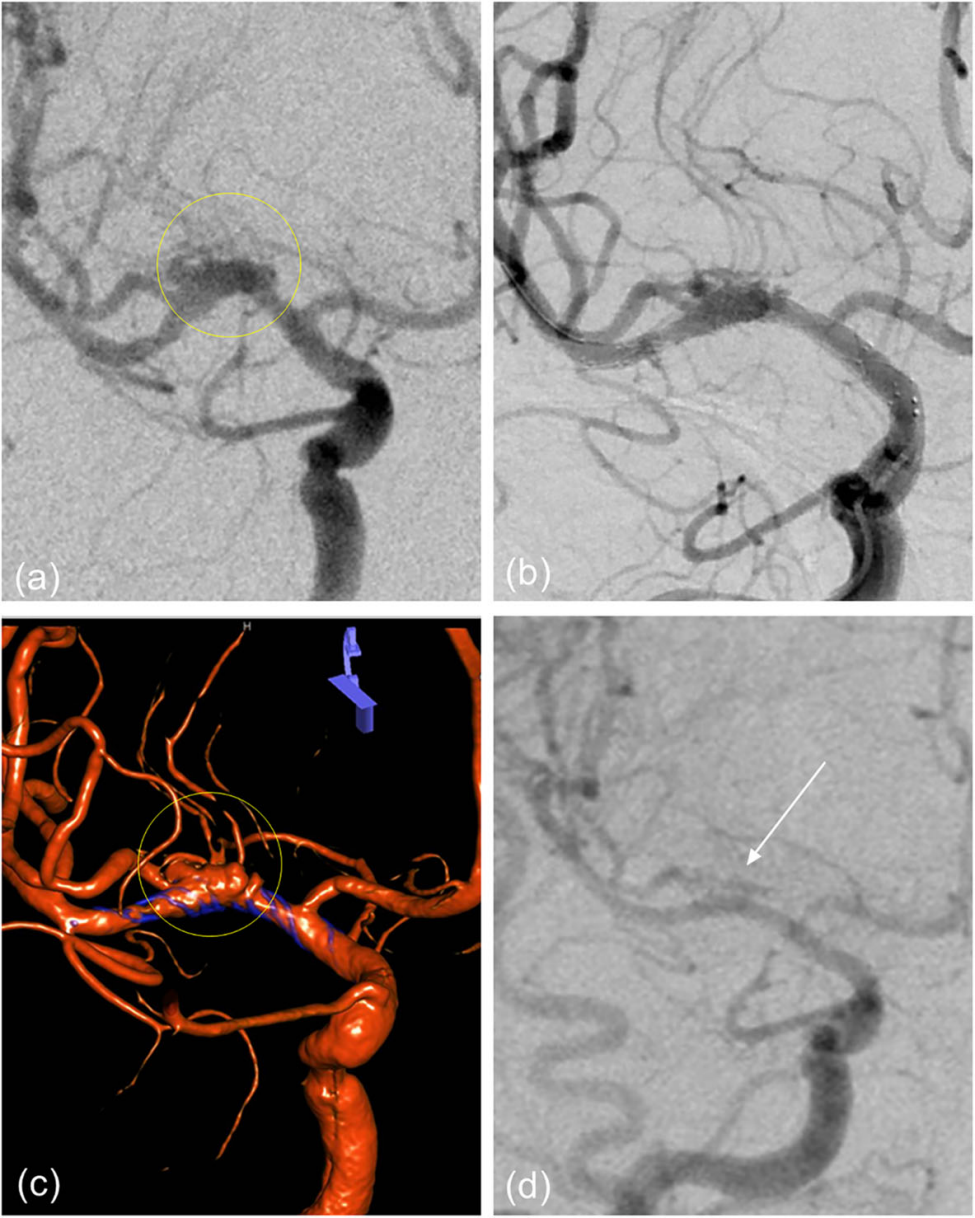
**a** The angiography of patient 5 revealed a fusiform aneurysm of the right middle cerebral artery, involving the entire trunk and all of lenticulostriate arteries. **b** We used telescopic stenting technology and implanted 2 LVIS stents (3.5×20mm, 3.5×15mm) in the right middle cerebral artery. **c** Postoperative angiography showed that the forward blood flow was satisfactory, and the lenticulostriate arteries were not affected. **d** Follow-up angiography after 5 months showed satisfactory repair of fusiform aneurysm

Six patients ranged in age from 43 to 66 years old. Half of the patients were male (*n* = 3, 50%). Each patient had a single aneurysm, and there were no aneurysms identified elsewhere in the intracranial circulation. The size of the aneurysms was listed in Table 1. According to the classification of our unique classification of piAN, one aneurysm were classified as type I, two as type IIa, two as type IIb, and the remaining one aneurysm was classified as type III. Two of the piANs were ruptured and the remaining four aneurysms were unruptured. All 6 patients received intravascular treatment, and the operations were very successful. Four patients with piANs had used coil-basket protection technology, and the other two patients had used the stent protection technology. All perforating arteries involved by piANs were successfully preserved. All patients had no cerebral hemorrhage or infarction caused by occlusion of perforating artery during perioperative period. Only one patient developed TIA symptoms 4 h after surgery, mainly manifested as motor aphasia and difficulty in expression. Postoperative head MRI revealed no infarcts. The symptoms were completely relieved in 24 h. There was no evidence of perforator infarction on the follow-up post-treatment imaging. The clinical follow-up time distribution of 6 patients ranges from 12 months to 50 months. Clinical follow-up data was available in 6 patients all achieving a good outcome (mRS ≤ 2) (100%). The results are summarized in Table 1, and demonstrated cases are showed in Figs. 2, 3, 4 and 5.

**Table 1:** Demographic, treatment, and follow-up data for each of the patients

Demographics			Aneurysm characteristics				Aneurysm state		Treatment		Post-op angiographic result		Follow-up		Complications	
Patient no.	Age	Gender	Size (mm)	Location	Perforating artery involving	Type	Rupture or not	Fisher’s grade	Technique type	Material used	Degree of aneurysm tamping	Protection of PA	Time	Angiographic result	Infarction	Repeat SAH
1	47	F	4.14×4.52 N:2.5	LC7	Anterior choroidal artery	IIa	Yes	2	②	LVIS 4.5*20 Coils 36.5cm	Satisfy	Yes	29M	Satisfy	No	No
2	43	M	10.0×10.8 N:10.0	BA bifurcation	BA perforating branches	IIb	Yes	1	②	Enterprise 4.5*28 Coils 181cm	Satisfy	Yes	26M	Satisfy	No	No
3	54	F	6.0×5.0 N:6.0	BA bifurcation	BA perforating branches	IIb	No	0	②	Enterprise 4.5*28 Coils 63cm	Satisfy	Yes	18M	Satisfy	No	No
4	54	F	2.0×3.3 N:1.41	Lenticulostriate arteries	Lenticulostriate arteries	I	No	0	①	LVIS 4.5*20 LVIS Jr 2.5*17 Coils 10cm	Satisfy	Yes	50M	Satisfy	No	No
5	66	M	L:20 W:5	RM1	Lenticulostriate arteries	III	No	0	①	LVIS*2 3.5*20 3.5*15	Satisfy	Yes	34M	Satisfy	No	No
6	58	M	3.2×4.1 N:4.1	LC7	Anterior choroidal artery	IIa	No	0	②	LVIS 3.5*20 Coils 27.5cm	Satisfy	Yes	13M	Satisfy	TIA	No

## Discussion

In this study, we proposed a unique three-classification method of piAN and perforator preservation technology. Six patients with piANs received endovascular therapy based on this new classification method and perforator preservation technology. All of 6 aneurysms obtained satisfactory obliteration, and all of their perforating arteries were successfully preserved. In terms of safety, only one patient developed an ischemic event in the territory of involving perforators 4 h after operation and completely resolved within 24 h. Follow-up angiography also showed patency of the parent artery and perforating arteries of treated aneurysms, with no aneurysmal recurrence.

Usually, treatment for intracranial aneurysms is done to achieve complete occlusion of the aneurysm without a remnant sac [7]. However, a large part of the complications in the treatment of aneurysms is due to the excessive pursuit of complete occlusion of the aneurysm and neglect of the protection of the peripheral perforating artery [8]. Such perforator involving aneurysms (piAN) are not uncommon in intracranial aneurysms due to the anatomical characteristics of intracranial vessels [1]. Pritz et al. found that perforators were present in 7% of basilar artery (BA) bifurcations, 17% of internal carotid artery (ICA) bifurcation aneurysms, 12% of middle cerebral aneurysms, and 11% of anterior communicating aneurysms [2]. Thus, preserving blood flow in the branches and perforators of a parent artery is very important for the successful treatment of piAN without postoperative morbidity and mortality [9, 10]. How to avoid this kind of situation is paid more and more attention by surgeons and constantly improve the surgical skills and methods to protect the blood supply of the perforating artery [11–13]. Sung-Pil Joo et al. discussed the consequences of perforator injury and how to avoid this phenomenon in aneurysm surgeries using intraoperative monitoring devices [1]. For surgical open craniotomy, the perforating artery can be observed under direct vision, and the damage to the perforating artery can be avoided by improving the shape of the aneurysm clip or the direction of the aneurysm clip.

However, there are still few scholars conducting research to propose how to protect the perforating artery in interventional treatment of intracranial aneurysms [14–16]. The advantage of interventional therapy is that it can intuitively observe the blood supply of the perforating artery. Our three-point classification is based on the anatomical relationship between the perforator artery and aneurysm which was first proposed. This classification method is more suitable for the endovascular treatment of piANs. We proposed two major perforator preservation technologies (PPT) based on this classification for the first time to help neurointerventional doctors to develop better surgical plans.

However, the classification and technologies we proposed still have certain flaws. Because we are still in the exploratory stage and the number of piAN cases enrolled in our center is still small, the two types of perforator preservation technology (PPT) based on our unique classification may not necessarily meet all piANs. Perhaps there are some complex piANs that cannot be classified in our proposed classification. Maybe some piANs need to combine two protection technologies and even need to design new perforator preservation technology to treatment. We proposed this unique three-classification method of piAN and perforator preservation technology and hoped to provide new ideas for the neurointerventionists. In the future case expansion and exploration, we hope to continuously improve the new classification method and perforator preservation technology to treat piANs.

## Conclusions

The primary results showed that our perforator preservation technologies on basis of the new neuro-interventional classification seem to be feasible, safe, and effective for endovascular treatment of perforator involving aneurysm. It helps to evaluate the surgical risk and design an appropriate surgical plan, and new ideas are provided for the interventional treatment of the perforator involving aneurysms.

## Data Availability

The datasets supporting the conclusion of this article are included within the article and its supplemental files.

## Abbreviations

PPT: Perforator preservation technology
piAN: Perforator involved aneurysms
ICA: Internal carotid artery
BAPAs: Basilar artery perforator aneurysms
LC7: The C7 segment of the left internal carotid artery
BA: Basilar artery
RM1: The M1 segment of the right middle cerebral artery
